# CTCF participates in DNA damage response via poly(ADP-ribosyl)ation

**DOI:** 10.1038/srep43530

**Published:** 2017-03-06

**Authors:** Deqiang Han, Qian Chen, Jiazhong Shi, Feng Zhang, Xiaochun Yu

**Affiliations:** 1 Department of Cancer Genetics and Epigenetics, Beckman Research Institute, City of Hope, 1500 E. Duarte Rd, Duarte, California, 91010, USA; 2 Department of Biochemistry and Molecular Biology, State Key Laboratory of Medical Molecular Biology, Institute of Basic Medical Sciences Chinese Academy of Medical Sciences, Peking Union Medical College, Beijing 100005, China; 3 Department of Cell Biology, Third Military Medical University, Chongqing, 400038, China

## Abstract

CCCTC-binding factor (CTCF) plays an essential role in regulating the structure of chromatin by binding DNA strands for defining the boundary between active and heterochromatic DNA. However, the role of CTCF in DNA damage response remains elusive. Here, we show that CTCF is quickly recruited to the sites of DNA damage. The fast recruitment is mediated by the zinc finger domain and poly (ADP-ribose) (PAR). Further analyses show that only three zinc finger motifs are essential for PAR recognition. Moreover, CTCF-deficient cells are hypersensitive to genotoxic stress such as ionizing radiation (IR). Taken together, these results suggest that CTCF participate in DNA damage response via poly(ADP-ribosylation).

Chromatin is dynamically regulated to higher order structures^1^,^2^. In response to DNA damage, a proper chromatin state is formed for facilitating DNA repair. Otherwise, it may cause genomic instability and result in human disorders, such as cancer^3^,^4^. Recent evidences showed that DNA-damage-induced chromatin modifications regulate the architecture of the chromatin at DNA lesions by recruiting chromatin remodeling factors^2^,^5^,^6^. One of the early chromatin modifications in response to DNA damage is poly (ADP-ribosyl)ation (PARylation)^7^,^8^.

PARylation is mediated by a group of poly (ADP-ribose) polymerases (PARPs), especially PARP1, the funding member of this family enzymes. In response to DNA damage, PARPs quickly catalyze PARylation at DNA lesions with NAD^+^ as ADP-ribose donor in this reaction. Within a few minutes following DNA damage, PARPs are able to use up to 90% cellular NAD^+^ to build massive PAR at DNA lesions. PAR not only brings huge amount of negative charges to relax the chromatin, but also is recognized by various types of PAR-binding motifs that mediate chromatin remodeling and facilitate DNA damage repair^9^,^10^,^11^,^12^,^13^,^14^,^15^. However, the underlying molecular mechanism of PAR-mediated chromatin remodeling remains elusive.

Once DNA damage occurs, DNA damage repair factors are recruited hierarchically at and surround the sites of DNA damage^16^,^17^. Their assembly at DNA damage sites involves complex spatial and temporal coordination of many dynamic interactions among repair proteins, and one key regulator during these processes is PARP1-depedent PARylation. We and others have shown that numerous DNA damage response factors recognize PARylation signals at DNA lesions, which mediates early and fast recruitment of these DNA damage response factors^10^,^18^. Over the past few years, many PAR-binding motifs have been identified, including the PBZ, MACRO, BRCT, FHA, RRM, OB-fold, PIN domains, in various DNA damage response factors^11^,^14^,^18^,^19^,^20^,^21^. This early and fast wave of the recruitment brings DNA damage response factors to the proximity of DNA lesions. With additional selection signals, such protein phosphorylation and ubiquitination, DNA damage response factors are anchored at DNA lesions for DNA damage repair^22^.

CTCF, a highly conserved nuclear polypeptide with 11 zinc finger motifs, is reported to bind to a variety of DNA loci and establish chromatin barriers between transcriptionally active and heterochromatic DNA^23^,^24^,^25^,^26^. Recent studies show that CTCF regulates three-dimensional structure of the chromatin fiber in the nucleus, based on its ability to mediate interactions between distant sequences^23^,^27^,^28^. With this unique feature, CTCF is involved in diversified regulatory functions, including promoter activation or repression, regulating transcriptional pausing and alternative mRNA splicing, methylation-dependent chromatin insulation, and genomic imprinting^29^,^30^,^31^. However, the molecular mechanism of CTCF’s recruitment to DNA lesions is still unclear.

In this study, we show that CTCF is quickly recruited to sites of DNA damage, which is mediated by PARylation. We have mapped the essential PAR-binding motif of CTCF. Thus, our study provides a novel regulation mechanism in DNA damage response.

## Results

### CTCF is quickly recruited to the sites of DNA damage

**Figure 1:**
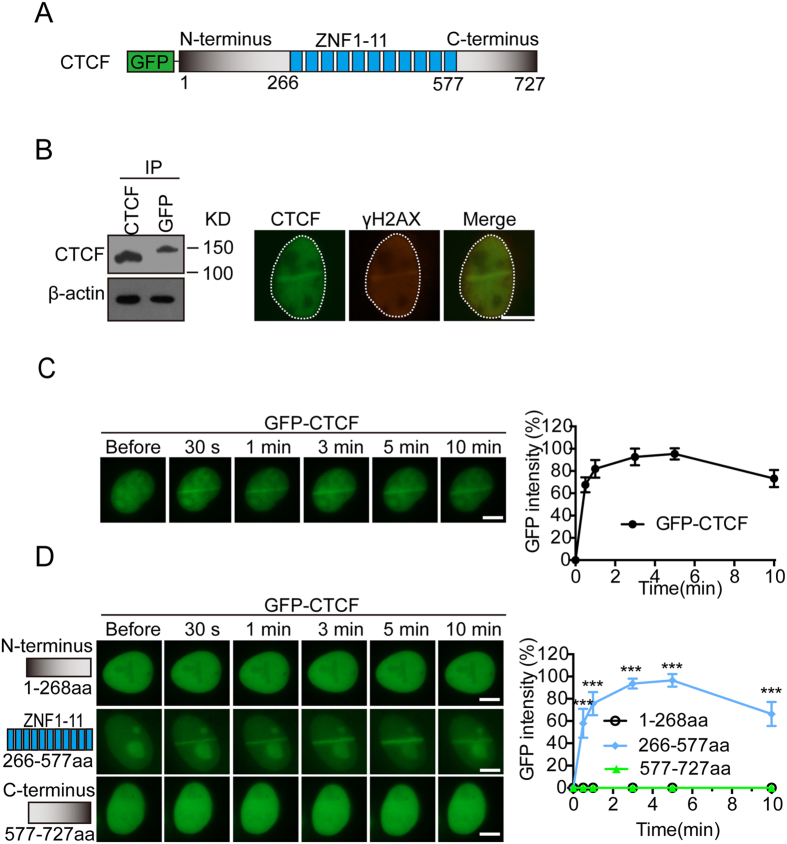
CTCF is quickly recruited to DNA lesions. (**A**) Schematic representation of the domain architecture of CTCF. (**B**) GFP-tagged CTCF (GFP-CTCF) co-localizes with γH2AX at laser irradiated DNA damage tracts. Left panel: Expression levels of exogenous and endogenous CTCF were measured by IP and Western blot with indicated antibodies. The CTCF blot was cropped and the full-length blot was included in the Fig. S5A. (**C**) The relocation kinetics of GFP-CTCF to DNA damage sites. GFP-tagged CTCF was expressed in U2OS cells, and the relocation kinetics was monitored in a time course following laser microirradiation. (**D**) GFP-tagged CTCF truncated mutants (N-terminus, C-terminus and ZNF1-11) were expressed in U2OS cells and the relocation kinetics was monitored in a time course following laser microirradiation. GFP signal intensities at the laser lines were converted into a numerical value using Image J software. Normalized fluorescent curves from 20 cells were averaged. Scale bar = 10 μm. The error bars represent the standard deviation. Significance of differences was evaluated by Student’s t test. ***p < 0.001. Signal intensities were plotted using GraphPad Prism software.

To investigate whether CTCF participates in DNA damage response, we ask if CTCF is recruited to the sites of DNA damage. We first generated a construct with a GFP tag at the N-terminus of CTCF and expressed it in U2OS cells (Fig. 1A). The GFP-CTCF was expressed in U2OS cells with lower expression level compared to the endogenous level of CTCF, and the cells were treated with laser microirradiation to induced DNA lesions. Interestingly, GFP-tagged CTCF was quickly recruited to the site of DNA damage and co-localized with γH2AX (Fig. 1B). We carefully measured the recruitment kinetics of CTCF and found that CTCF reached to DNA lesions within 30 seconds, indicating that CTCF may participate in early events during DNA damage response (Fig. 1C). To examine which domain in CTCF mediates such quick recruitment, we dissect each domain by generating truncation mutants of CTCF. Since CTCF includes an N-terminal region, a zinc finger domain with 11 zinc finger motifs, a C-terminal region. We fused GFP to each truncation mutant and found that only the zinc finger domain, but not the N or C-terminal regions, was able to reach the sites of DNA damage (Fig. 1D). Moreover, similar to that of the full length of CTCF, the zinc finger domain itself was quickly recruited to DNA lesions within 30 seconds, indicating that the zinc finger domain is responsible for the early recruitment of CTCF to DNA lesions.

### PAR mediates the early recruitment of CTCF to DNA lesions

**Figure 2:**
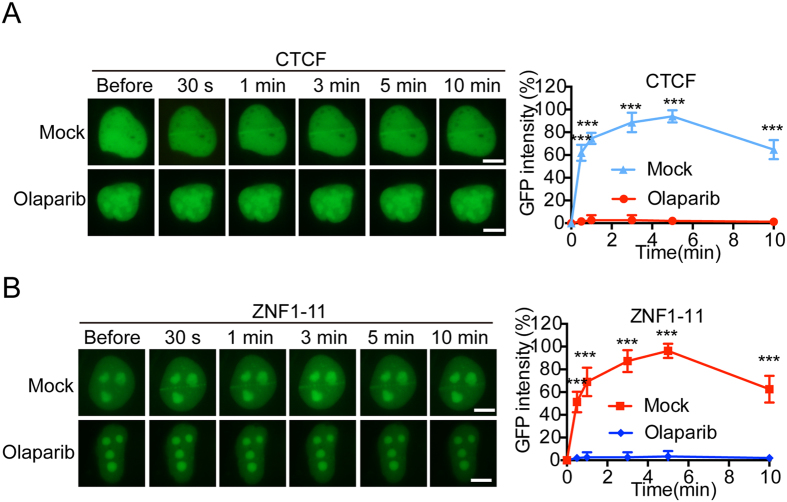
PAR mediates the early recruitment of CTCF to DNA lesions. (**A**) Olaparib treatment suppresses the recruitment of GFP-CTCF to DNA damage sites. (**B**) The effect of Olaparib treatment on the recruitment of GFP-CTCF-ZNF1-11 to DNA damage sites. GFP-CTCF (**A**) or GFP-ZNF1-11 (**B**) was expressed in U2OS and treated with 1 μM Olaparib for 1 hour. The relocation was monitored in a time course following laser microirradiation. GFP signal intensities at the laser line were converted into a numerical value using Image J software. Normalized fluorescent curves from 20 cells were averaged. Scale bar = 10 μm. The error bars represent the standard deviation. Significance of differences was evaluated by Student’s t test. *** *p* < 0.001. Signal intensities were plotted using GraphPad Prism software.

Next, we sought what mediates the recruitment of CTCF to DNA lesions. Since the relocation kinetics of CTCF to DNA damage sites is very similar to that of PARylation, one of earliest signals generated at DNA lesions, we ask if PARylation mediates the early recruitment of CTCF. We treated the U2OS cells expressing GFP-CTCF with Olaparib, a potent PARP inhibitor to suppress PAR synthesis, and found that the recruitment of CTCF was remarkably abolished (Fig. 2A). Since the zinc finger domain mediates the recruitment, we also ask if PAR is required for the recruitment of the zinc finger domain of CTCF. Similar to the full length of CTCF, the recruitment of the zinc finger domain was abolished with the treatment of Olaparib as well (Fig. 2B). To exclude the possible off-target effect of Olaparib, we verified the recruitment of CTCF in the PARP1 null U2OS cells. Again, the results showed that the CTCF recruitment was disrupted in the PARP1 null U2OS cells (Fig. S1). Collectively, these results suggest that PARylation is required for the early recruitment of CTCF to DNA lesions.

### CTCF directly binds to PAR

**Figure 3:**
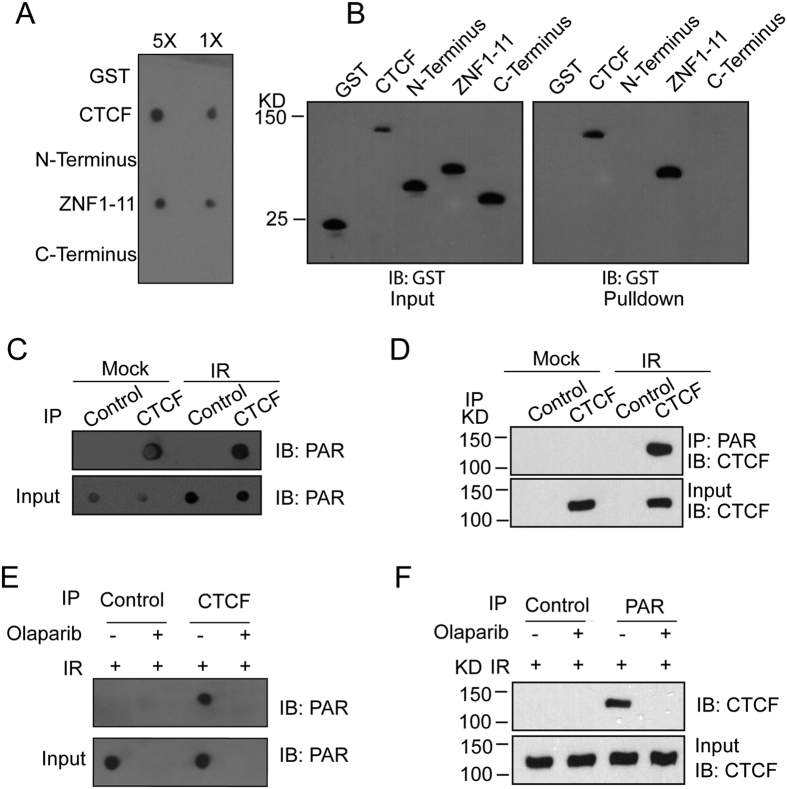
CTCF directly binds to PAR. (**A**) The recombinant GST-fusion proteins were incubated with PAR. Protein-associated PAR was examined by glutathione agarose beads pull down and dot blotting with anti-PAR antibody. Recombinant GST was used as the negative controls. (**B**) CTCF and ZNF1-11 interact with PAR. Left panel: the recombinant GST-CTCF and CTCF truncated mutants (N-terminus, C-terminus and ZNF1-11) were incubated with or without biotin-PAR. The interaction was examined by streptavidin beads pull-down and Western blotting with anti-GST antibody. GST was used as the negative controls. (**C**) The *in vivo* interaction between CTCF and PAR was examined by co-IP and reciprocal co-IP (**D**). U2OS cells were treated with 0 or 10 Gy of IR. 5 min after IR, cells were lysed and analyzed with indicated antibodies. Samples of input or IP were analyzed by dot blotting (**C**) or Western blotting (**D**) with the indicated antibodies. The CTCF blot was cropped and the full-length blot was included in the Fig. S5C. (**E,F**) The *in vivo* interaction between CTCF and PAR was examined by co-IP and reciprocal co-IP in the presence or absence of Olaparib (100 nM) and IR treatment (10 Gy) with the indicated antibodies. Irrelevant IgG was included as the IP control. The CTCF blot was cropped and the full-length blot was included in the Fig. S5D.

The fast recruitment by PARylation indicates that CTCF recognizes PAR at DNA lesions. Since the zinc finger domain of CTCF targets the relocation, we ask if the zing finger domain is a PAR-binding module. We first synthesized and purified PAR, and performed an *in vitro* binding assay by incubating the recombinant GST-CTCF with PAR. The pull down assays show that GST-CTCF but not GST protein bound to PAR. Similar to full length CTCF, the zinc finger domain of CTCF was sufficient to bind PAR (Fig. 3A). Moreover, in the reciprocal pull down assays, PAR was able to pull down full length CTCF as well as the zinc finger domain of CTCF (Fig. 3B). Taken together, these results suggest that the zinc finger domain mediates PAR-binding.

To examine if CTCF interacts with PAR *in vivo*, we performed immunoprecipitation (IP) with anti-CTCF antibody and dot blotting assays with anti-PAR antibody. We found that CTCF associated with PAR *in vivo*, especially when PAR was massively synthesized following DNA damage (Fig. 3C). This interaction was further confirmed by reciprocal IP (Fig. 3D). Moreover, we treated cells with Olaparib to suppress PAR synthesis and found that CTCF no longer interacted with PAR following DNA damage (Fig. 3E and F). Taken together, these results suggest that CTCF recognizes PAR *in vivo*, especially following DNA damage.

### Specific zinc finger motifs are essential to mediate the recruitment of CTCF

**Figure 4:**
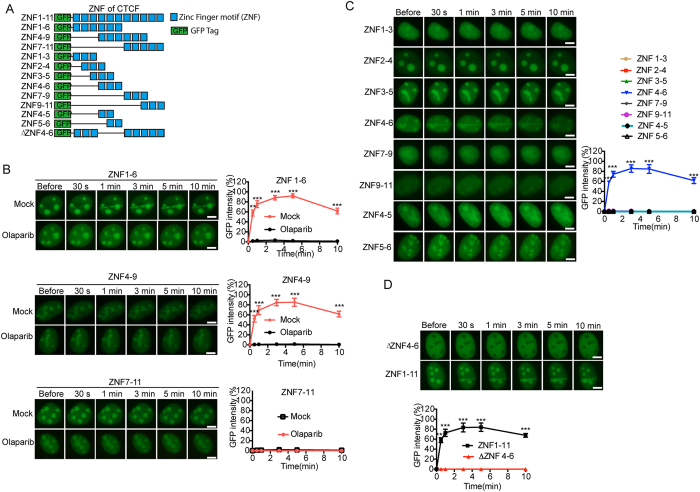
Specific zinc finger motifs are essential to mediate the recruitment of CTCF. (**A**) Schematic representation of the truncated ZNF. (**B**) The effect of Olaparib treatment on the recruitment of ZNF1-6, ZNF4-9 and ZNF7-11. GFP-ZNF1-6, GFP-ZNF4-9 and GFP-ZNF7-11 were expressed in U2OS cells treated with or without 1 μM Olaparib. The relocation kinetics was examined. (**C**) The ZNF 4-6 of CTCF is sufficient for the relocation to DNA lesions. GFP-ZNF1-3, ZNF2-4, ZNF3-5, ZNF4-6, ZNF7-9, ZNF9-11, ZNF4-5, ZNF5-6 were expressed in U2OS. The relocation was kinetics was examined. (**D**) Deletion of ZNF4-6 abolishes the relocation. ZNF1-11 was used a positive control. Normalized fluorescent curves from 20 cells were averaged. The error bars represent the standard deviation. Significance of differences was evaluated by Student’s t test. **p < 0.01 and ***p < 0.001. Signal intensities were plotted using GraphPad Prism software. Scale bar = 10 μm.

CTCF contains conserved 11 zinc finger motifs. To assess which exact zinc finger motif(s) is essential for the recruitment of CTCF to DNA lesions, we generated various truncation mutants and fused them with a GFP tag, and investigated the kinetics of relocation of these mutants (Fig. 4A). We first generated three large truncation mutants including ZNF1-6, ZNF4-9 and ZNF7-11. Both ZNF1-6 and ZNF4-9, but not ZNF7-11, were recruited to DNA damage site quickly (Fig. 4B). Moreover, to define the minimal region that mediates the relocation of CTCF, we generated second round truncation mutants including ZNF1-3, ZNF2-4, ZNF3-5, ZNF4-6, ZNF7-9 and ZNF9-11. Only ZNF4-6, but not other zinc finger motifs, was able to relocate to the sites of DNA damage (Fig. 4C). However, further deletion of any zinc finger motif inside of ZNF4-6 abolished the recruitment, suggesting that all three zinc finger motif are essential for the recruitment. Moreover, we confirmed the results by deletion of ZNF4-6 from the whole zinc finger domain, which abolished the recruitment (Fig. 4D). Thus, these results indicate that CTCF recruited to DNA damage sites by the minimal region with at least three zinc fingers.

### ZNF4-6 recognizes PAR

**Figure 5:**
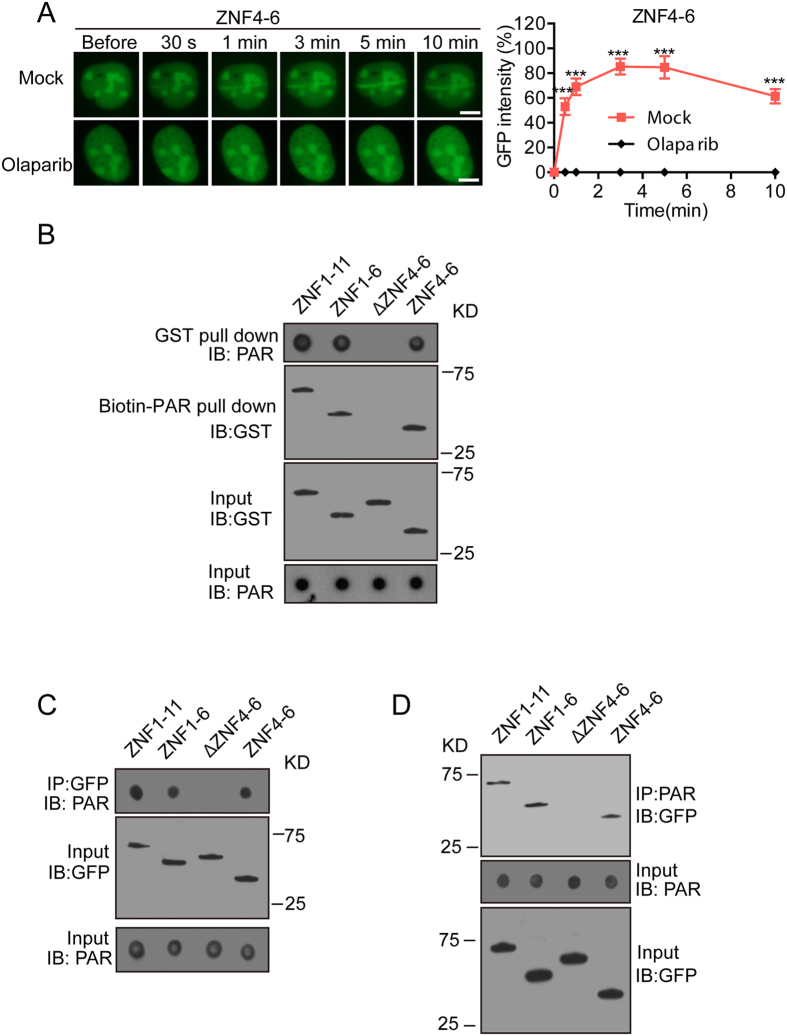
ZNF4-6 of CTCF recognizes PAR. (**A**) The effect of Olaparib treatment on the recruitment of GFP-ZNF4-6 to DNA damage sites. (**B**) Pull down assays were performed to detect the interaction between GST-ZNF1-11, ZNF1-6, ΔZNF4-6, ZNF4-6 and biotin-PAR. The recombinant mutants of CTCF-ZNF were incubated with biotin-PAR. The interaction was either examined by streptavidin beads pull-down assay and western blotting with anti-GST antibody or was examined by Glutathione Sepharose pull-down and western blotting with Streptavidin-HRP. The interaction between GFP-ZNF1-11, ZNF1-6, ΔZNF4-6, ZNF4-6 and PAR was examined by co-IP (**C**) and reciprocal co-IP (**D**). The blots were cropped and the full-length blots were included in the Fig. S5E, F and G.

Since ZNF4-6 is able to relocate to DNA lesions, we ask if ZNF4-6 recognizes PAR. We first treated the cells with PARPs inhibitor Olaparib and found that the recruitment of ZNF4-6 was abolished, suggesting that the recruitment is likely mediated by PAR (Fig. 5A). We next performed an *in vitro* binding assay by incubating PAR and recombinant ZNF1-11, ZNF1-6, ΔZNF4-6 and ZNF4-6, respectively. The pull-down results show that ZNF4-6 is sufficient to bind PAR, and loss of ZNF4-6 (ΔZNF4-6) abolishes the binding with PAR (Fig. 5B). Similar results were obtained using a reverse pull-down assay (Fig. 5B), suggesting that ZNF4-6 is the minimal PAR-binding module inside of CTCF. We also performed co-IP and dot blotting assays with anti-PAR and anti-GFP antibodies, and confirmed that ZNF4-6 recognized PAR *in vivo* (Fig. 5C and D).

Besides PAR-binding, it has been shown that the zinc finger domain of CTCF recognizes specific DNA sequence to establish boundary between euchromatin and heterochromatin for higher order of nuclear architecture. Here, we sought to determine whether PAR-binding of CTCF interferes its DNA binding. We performed a gel mobility shift analysis by incubating the recombinant GST, ZNF or ΔZNF4-6 with ^32^P-labeled DNA with conserved sequence of insulator^32^,^33^,^34^,^35^. Not only ZNF1-11 but ΔZNF4-6 bound DNA, indicating that PAR-binding module is not essential for DNA-binding (Fig. S2A). Moreover, the affinity between insulator DNA and ΔZNF4-6 was only slightly reduced compared to that between full length zinc finger domain and insulator DNA (Fig. S2B). The results suggest that although ZNF4-6 might facilitate DNA binding, other zinc finger motifs (e.g. ZNF1-4 and 7-11) are able to largely compensate the loss of ZNF4-6 for DNA binding.

### CTCF participates in DNA damage response

**Figure 6:**
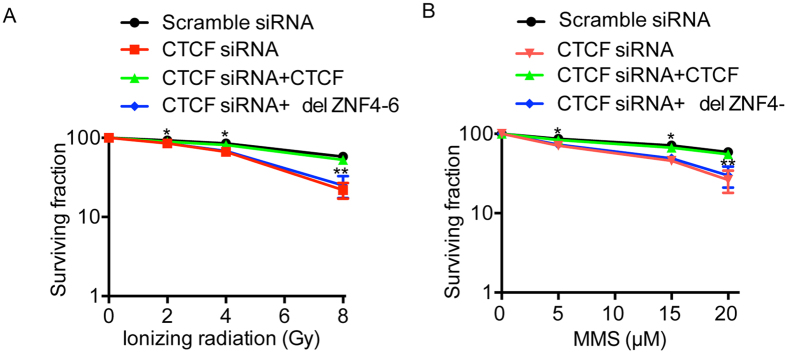
CTCF participates in DNA damage repair. Cells lacking CTCF are hypersensitive to DNA damaging agents. U2OS cells were treated with indicated siRNA and/or expressing indicated proteins before IR treatment (**A**), or MMS treatment (**B**). Significance of differences was evaluated by Student’s t test. *p < 0.05 and **p < 0.01.

The relocation of CTCF to the sites of DNA damage indicates that CTCF participates in DNA damage response. To test this hypothesis, we depleted CTCF using siRNA in U2OS cells, and treated the cells with IR and MMS. The expression level of CTCF protein in the CTCF knockdown cells was dramatically decreased (Fig. S3). Interestingly, cells lacking CTCF were hypersensitive to both IR and MMS (Fig. 6A and B), both of which are able to induce massive PARylation^36^. Moreover, reconstituting the cells with full length CTCF but not ZNF4-6 deletion mutant (del ZNF4-6) rescued the cellular phenotype (Fig. 6A and B), suggesting that PAR-mediated relocation of CTCF plays an important role for DNA damage response.

## Discussion

Our study provides the first evidence that CTCF is recruited to DNA lesions, suggesting that CTCF participates in DNA damage response. The recruitment is mediated by its zinc finger domain and PAR. Since PAR is one of the early signals at the sites of DNA damage^7^,^19^, CTCF is quickly recruited to DNA lesions within 30 seconds. This early recruitment is similar to the recruitment of other zinc finger motif-containing proteins, such as Chfr^21^. We and others have shown that similar to CTCF, Chfr also uses the zinc finger motifs to recognize PAR, which mediates early recruitment of Chfr to the site of DNA lesions^21^,^37^. Different from Chfr, CTCF has 11 zinc finger motifs. Only zinc finger motif 4–6 mediates the binding of PAR. It has been shown that the zinc finger domain of CTCF binds DNA as well^23^,^38^. Here, we found that lacking zinc finger motif 4–6 did not significantly weaken the DNA-binding. However, the binding with PAR was largely abolished by ΔZNF4–6, suggesting that CTCF may use different zinc finger motifs to recognize different functional partners. Structurally, poly (ADP-ribose) is similar to single-stranded DNA. Both DNA and PAR contains ribose, phosphate group and adenine. It would be intriguing to examine the molecular details of the PAR-binding and DNA-binding of CTCF with structural analysis in future.

The early recruitment of CTCF indicates the early DNA damage response mediated by CTCF. It has been shown that CTCF is a chromatin barrier defines the boundaries between euchromatin and heterochromatin^23^,^39^. Following DNA damage, DNA damage response boundary is also formed to prevent the extensive spreading of the damage signals^22^. Here, we measured the IR-induced foci formation (IRIF) of γH2AX, a surrogate marker of DNA double-strand breaks. Although CTCF did not affect the IRIF formation of γH2AX, lacking CTCF slightly increase the foci quantity in each cell as well as the number of foci positive cells (Fig. S4A and B). More interestingly, we found that the average size of each focus might be increased in cells lacking CTCF, suggesting that CTCF might play a role for controlling the expansion of DNA damage response region (Fig. S4C and D). Moreover, reconstitution siRNA treated cells with full length CTCF restored the size of IRIF of γH2AX, whereas the cells only expressing del ZNF4-6 failed to rescue the cellular phenotype (Fig. S4C and D). Limiting the DNA damage response may protect the high order structure of adjacent chromatin and facilitate gene transcription in non-DNA damage regions^22^. In particular, DNA damage-induced transcription of DNA damage repair genes plays a key role for lesion repair^40^. Thus, loss of the boundary may suppress DNA damage repair and induce cell lethality. Consistently, we found that CTCF is important for cell survival following DNA damage. Although the size of IRIF of γH2AX was significantly increased in the absence of CTCF, γH2AX was still limited to a certain compartment. It is possible that besides CTCF, other factors or other layers of regulation may also play roles to limit DNA damage response. In particular, the size of IRIF of γH2AX is still growing during DNA damage repair even in the presence of CTCF. Thus, it is likely that CTCF is only required for the early limitation of DNA damage response. And other factors may play important role for the regulation of late DNA damage response. Consistently, CTCF is recruited by PARylation, an early DNA damage response signals.

Beside the establishment of the boundary, it has been reported that CTCF is important for the formation of higher-order chromatin structure, such as maintaining the three-dimensional organization of chromatin^28^. Genomes DNA is packaged into multiple levels of organization dynamically, from nucleosomes to chromosome when DNA damage occurs^3^. Thus, it is possible that loss of CTCF abolishes higher-order chromatin structure and affects the DNA damage repair. Nevertheless, our study demonstrates that CTCT is quickly recruited to the sites of DNA damage by PARylation and plays an important role in early DNA damage response.

## Materials and Methods

### Plasmids, siRNA and CRISPR/Cas9 sgRNA Sequence

Human CTCF full length cDNA (ACCESSION BT009915), N-terminus (1–268 aa), zinc finger domain (266–577 aa), C-terminus (577–727 aa) were cloned into pEGFP-C1 or pGEX-4T-1 vectors. Deletion mutants of pEGFP-C1/ΔZNF4-6 or pGEX-4T-1/ΔZNF4-6 were constructed by site-directed mutagenesis. All the PCR products were digested with the restriction enzymes EcoRI and SalI and cloned into EcoRI- and SalI-digested (NEB) null plasmid vector. Single Zinc finger domain was cloned into pEGFP-C1 with nuclear localization sequence (NLS) at N terminus. NLS sequence is CCGAAGAAGAAGCGGAAGGTT. ON-TARGET plus CTCF siRNA (L-020165-00-0005) were purchased from Dharmacon. The siRNA were transfected into cells using Oligofectamine (Invitrogen) according to manufacturer’s instructions. pSpCas9(BB)-2A-Puro (PX459) V2.0 was purchased from Addgene (plasmid #62988). The single guide RNA (sgRNA) sequence for PARP1 used in this study was 5′-CCACCTCAACGTCAGGGTGC-3′.

### Cell Culture, antibody, immunoprecipitation and Western blotting

U2OS or 293 T cells were maintained in DMEM medium with 10% fetal bovine serum and cultivated at 37 °C in 5% CO_2_ (v/v). U2OS cells were transfected with PX459 vector containing PARP1-sgRNA for PARP1 knockout. Transfected cells were plated at low density in 1.5 mg/ml puromycin (Invitrogen). Single colonies were propagated, and individual clones were assessed by western blotting. Loss of PARP1 in U2OS cells was validated by anti-PARP1 antibody which was purchased from Cell Signaling Technology (Cat# 9542).

Cells were lysed with NETN buffer (0.5% NP40, 50 mM Tris-HCl pH 8.0, 100 mM NaCl, 2 mM EDTA) with Roche Protease Inhibitor Cocktail. Immunoprecipitation and Western blotting were performed following standard protocol as described previously^20^. Rabbit anti-CTCF antibody was purchased from Cell Signaling. Monoclonal anti-PAR antibody was purchased from Trevigen. Mouse monoclonal anti-GFP antibody was purchased from Cell Signaling.

### Gel mobility shift analysis

The affinity binding between the ZNF or mutants of CTCF and DNA was measured by gel mobility shift assays. 1 pmol of each of the purified proteins were incubated with 0.5 pmol of the ^32^P-labeled duplex oligonucleotide (CCCCCAGGGATGTAATTACGTCCCTCCCCCGCTAGGGGGCAGCAG) in HEPEs buffer (25 mM HEPES pH 8.0, 50 mM KCl, 50 mM MgCl_2_, 1% NP40, 10% glycerol and 200 ng poly (dI/dC)) for 20 min at 4 °C. The samples were analyzed with 7.5% polyacrylamide gel (Acrylamide: Bis-Acrylamide 29:1, 40% Solution, Thermo Fisher Scientific).

### Synthesis and purification of PAR and biotin-PAR

PAR was synthesized and purified as described previously^41^. His-PARP1 was expressed in BL21 and purified by Ni-NTA affinity resin. PAR was synthesized in a 1 ml incubation buffer containing 100 mM Tris-HCl pH 7.8, 10 mM MgCl_2_, 1 mM NAD^+^, 10 mM DTT, 1 mg histone, 50 μg octameric oligonucleotide GGAATTCC and 100 μg recombinant PARP1. To generate biotinyl-PAR, 1 μM biotinyl-NAD^+^ (Trevigen) was included in the reaction. The mixture was incubated at 37 °C for 60 min and stopped by an addition of 20 ml ice-cold 20% TCA. Oligo DNA was removed by DNase I and proteins were digested by proteinase K. Procedure for PAR Purification was described previously.

### PAR binding assays

Approximately 5 pmol PAR and 1 pmol (5X) or 0.2 pmol (1X) of each recombinant protein were incubated together with 30 μl glutathione agarose in PBS. After incubation for 1 h at room temperature, beads were extensively washed with PBS 3 times, and bound proteins were released by adding 30 μl sample buffer followed by heating at 90 °C for 8 min. 2 μl aliquots of samples were dotted onto nitrocellulose membranes. After incubation for 20 min at 60 °C, dot blotting assays were performed with anti-PAR antibody.

### GST fusion protein expression and pull-down assay

GST fusion proteins were expressed in E. coli BL21 and purified using standard procedures. Purified GST fusion proteins (0–40 pmol) were incubated with biotin-labeled PAR (10 pmol) and streptavidin beads for 2 h at 4 °C. After washing with NETN-100 buffer for four times, samples were boiled in SDS sample buffer and elutes were analyzed by Western blotting with anti-GST antibody.

### Laser microirradiation and microscope image acquisition

Cells transfected with GFP-tagged corresponding plasmids were grown on 35-mm glass bottom dishes (Corporation). Laser microirradiation was performed on OLYMPUS IX71 inverted fluorescence microscope coupled with the MicroPoint Laser Illumination and Ablation System (Photonic Instruments, Inc.). A 337.1 nm laser diode (3.4 mW) transmits through a specific Dye Cell and then yields 365 nm wavelength laser beam that is focused through X60 UPlanSApo / 1.35 oil objective to yield a spot size of 0.5–1 μm The pulse energy is 170 μJ at 10 Hz. Images were taken by the same microscope with CellSens software (Olympus). The GPF fluorescence strips were recorded at indicated time points and then analyzed with Image J software. 20 cells were analyzed from three independent experiments. Error bars represent the standard deviation.

### Immunofluorescence staining

For immunofluorescence staining, cells were treated with laser microirradiation or different dose of IR, and were washed 3 times by PBS. Then cells were fixed in 3% paraformaldehyde for 5 min and permeabilized with 0.5% Triton X-100 in phosphate-buffered saline (PBS) for 5 min at room temperature. Samples were blocked with 8% goat serum and then incubated with the primary antibody for 1 h. Samples were washed for three times and incubated with the secondary antibody for 45 min. Follow washing 3 times by PBS, sample were added 100 μl DAPI. Then the coverslips were mounted onto glass slides and visualized with OLYMPUS IX71 inverted fluorescence microscope. For the analysis of IRIF, 100 cells in each sample were analyzed with Image J software. Monoclonal anti-phospho-Histone H2AX (Ser139) antibody was purchased from EMD Millipore (Cat# 05–636).

### Cell survival assays

One thousand cells were transfected with scramble siRNA or CTCF siRNAs. Cells were seeded onto 60 mm dishes. Twenty-four hours after transfection, cells were treated with irradiated or MMS. Medium was replaced 1 h later and cells were then incubated for 10 days. The surviving cell fractions were calculated by comparing the numbers of colonies formed in the cultures plates. For rescue assay, U2OS transfected with CTCF siRNA for 48 hours, and then transfected with plasmids encoding full length CTCF or mutants.

### Statistical analysis

Data are represented as mean ± SD as indicated from at three independent experiments. Significance of differences was evaluated by Student’s t test. *p* < 0.05 was considered as statistically significant.

## Additional Information

**How to cite this article:** Han, D. *et al*. CTCF participates in DNA damage response via poly(ADP-ribosyl)ation. *Sci. Rep.* **7**, 43530; doi: 10.1038/srep43530 (2017).

**Publisher's note:** Springer Nature remains neutral with regard to jurisdictional claims in published maps and institutional affiliations.

## Supplementary Material

Supplemental Data

## References

[b1] Kornberg R. D. Chromatin structure: a repeating unit of histones and DNA. Science (New York, N.Y.) 184, 868–871 (1974). 10.1126/science.184.4139.868 4825889

[b2] Varga-Weisz P. D. & Becker P. B. Regulation of higher-order chromatin structures by nucleosome-remodelling factors. Current opinion in genetics & development 16, 151–156 (2006). 16503135 10.1016/j.gde.2006.02.006

[b3] Price Brendan D. & D’Andrea Alan D. Chromatin Remodeling at DNA Double-Strand Breaks. Cell 152, 1344–1354 (2013). 23498941 10.1016/j.cell.2013.02.011 PMC3670600

[b4] Sulli G., Di Micco R. & di Fagagna F. d. A. Crosstalk between chromatin state and DNA damage response in cellular senescence and cancer. Nat Rev Cancer 12, 709–720 (2012). 22952011 10.1038/nrc3344

[b5] van Attikum H. & Gasser S. M. Crosstalk between histone modifications during the DNA damage response. Trends in cell biology 19, 207–217 (2009). 19342239 10.1016/j.tcb.2009.03.001

[b6] Kouzarides T. Chromatin modifications and their function. Cell 128, 693–705 (2007). 17320507 10.1016/j.cell.2007.02.005

[b7] Schreiber V., Dantzer F., Ame J. C. & de Murcia G. Poly(ADP-ribose): novel functions for an old molecule. Nature reviews. Molecular cell biology 7, 517–528 (2006). 16829982 10.1038/nrm1963

[b8] Hassa P. O. & Hottiger M. O. The diverse biological roles of mammalian PARPS, a small but powerful family of poly-ADP-ribose polymerases. Frontiers in bioscience : a journal and virtual library 13, 3046–3082 (2008). 17981777 10.2741/2909

[b9] Kim M. Y., Zhang T. & Kraus W. L. Poly(ADP-ribosyl)ation by PARP-1: ‘PAR-laying’ NAD+ into a nuclear signal. Genes & development 19, 1951–1967 (2005). 16140981 10.1101/gad.1331805

[b10] Luo X. & Kraus W. L. On PAR with PARP: cellular stress signaling through poly(ADP-ribose) and PARP-1. Genes & development 26, 417–432 (2012). 22391446 10.1101/gad.183509.111 PMC3305980

[b11] Ahel I. . Poly(ADP-ribose)-binding zinc finger motifs in DNA repair/checkpoint proteins. Nature 451, 81–85 (2008). 18172500 10.1038/nature06420

[b12] Li G. Y. . Structure and identification of ADP-ribose recognition motifs of APLF and role in the DNA damage response. Proceedings of the National Academy of Sciences of the United States of America 107, 9129–9134 (2010). 20439749 10.1073/pnas.1000556107 PMC2889080

[b13] Li M., Bian C. & Yu X. Poly(ADP-ribosyl)ation is recognized by ECT2 during mitosis. Cell cycle (Georgetown, Tex.) 13, 2944–2951 (2014). 10.4161/15384101.2014.947197 PMC4614318 25486481

[b14] Zhang F., Chen Y., Li M. & Yu X. The oligonucleotide/oligosaccharide-binding fold motif is a poly(ADP-ribose)-binding domain that mediates DNA damage response. Proceedings of the National Academy of Sciences of the United States of America 111, 7278–7283 (2014). 24799691 10.1073/pnas.1318367111 PMC4034225

[b15] Sousa F. G. . PARPs and the DNA damage response. Carcinogenesis 33, 1433–1440 (2012). 22431722 10.1093/carcin/bgs132

[b16] Ciccia A. & Elledge S. J. The DNA damage response: making it safe to play with knives. Molecular cell 40, 179–204 (2010). 20965415 10.1016/j.molcel.2010.09.019 PMC2988877

[b17] Jackson S. P. & Bartek J. The DNA-damage response in human biology and disease. Nature 461, 1071–1078 (2009). 19847258 10.1038/nature08467 PMC2906700

[b18] Li M. & Yu X. Function of BRCA1 in the DNA damage response is mediated by ADP-ribosylation. Cancer cell 23, 693–704 (2013). 23680151 10.1016/j.ccr.2013.03.025 PMC3759356

[b19] Gibson B. A. & Kraus W. L. New insights into the molecular and cellular functions of poly(ADP-ribose) and PARPs. Nature reviews. Molecular cell biology 13, 411–424 (2012). 22713970 10.1038/nrm3376

[b20] Zhang F., Shi J., Chen S. H., Bian C. & Yu X. The PIN domain of EXO1 recognizes poly(ADP-ribose) in DNA damage response. Nucleic acids research 43, 10782–10794 (2015). 26400172 10.1093/nar/gkv939 PMC4678857

[b21] Liu C., Wu J., Paudyal S. C., You Z. & Yu X. CHFR is important for the first wave of ubiquitination at DNA damage sites. Nucleic acids research 41, 1698–1710 (2013). 23268447 10.1093/nar/gks1278 PMC3561977

[b22] Bekker-Jensen S. . Spatial organization of the mammalian genome surveillance machinery in response to DNA strand breaks. The Journal of cell biology 173, 195–206 (2006). 16618811 10.1083/jcb.200510130 PMC2063811

[b23] Ong C. T. & Corces V. G. CTCF: an architectural protein bridging genome topology and function. Nature reviews. Genetics 15, 234–246 (2014). 10.1038/nrg3663 PMC4610363 24614316

[b24] Baniahmad A., Steiner C., Kohne A. C. & Renkawitz R. Modular structure of a chicken lysozyme silencer: involvement of an unusual thyroid hormone receptor binding site. Cell 61, 505–514 (1990). 2159385 10.1016/0092-8674(90)90532-j

[b25] Lobanenkov V. V. . A novel sequence-specific DNA binding protein which interacts with three regularly spaced direct repeats of the CCCTC-motif in the 5′-flanking sequence of the chicken c-myc gene. Oncogene 5, 1743–1753 (1990). 2284094

[b26] Cuddapah S. . Global analysis of the insulator binding protein CTCF in chromatin barrier regions reveals demarcation of active and repressive domains. Genome research 19, 24–32 (2009). 19056695 10.1101/gr.082800.108 PMC2612964

[b27] Schmidt D. . Waves of retrotransposon expansion remodel genome organization and CTCF binding in multiple mammalian lineages. Cell 148, 335–348 (2012). 22244452 10.1016/j.cell.2011.11.058 PMC3368268

[b28] Nichols M. H. & Corces V. G. A CTCF Code for 3D Genome Architecture. Cell 162, 703–705 (2015). 26276625 10.1016/j.cell.2015.07.053 PMC4745123

[b29] Kurukuti S. . CTCF binding at the H19 imprinting control region mediates maternally inherited higher-order chromatin conformation to restrict enhancer access to Igf2. Proceedings of the National Academy of Sciences 103, 10684–10689 (2006). 10.1073/pnas.0600326103 PMC1484419 16815976

[b30] Phillips J. E. & Corces V. G. CTCF: master weaver of the genome. Cell 137, 1194–1211 (2009). 19563753 10.1016/j.cell.2009.06.001 PMC3040116

[b31] Ohlsson R., Renkawitz R. & Lobanenkov V. CTCF is a uniquely versatile transcription regulator linked to epigenetics and disease. TRENDS in Genetics 17, 520–527 (2001). 11525835 10.1016/s0168-9525(01)02366-6

[b32] Renda M. . Critical DNA binding interactions of the insulator protein CTCF: a small number of zinc fingers mediate strong binding, and a single finger-DNA interaction controls binding at imprinted loci. The Journal of biological chemistry 282, 33336–33345 (2007). 17827499 10.1074/jbc.M706213200

[b33] Vostrov A. A. & Quitschke W. W. The zinc finger protein CTCF binds to the APBbeta domain of the amyloid beta-protein precursor promoter. Evidence for a role in transcriptional activation. The Journal of biological chemistry 272, 33353–33359 (1997). 9407128 10.1074/jbc.272.52.33353

[b34] Engel N., West A. G., Felsenfeld G. & Bartolomei M. S. Antagonism between DNA hypermethylation and enhancer-blocking activity at the H19 DMD is uncovered by CpG mutations. Nature genetics 36, 883–888 (2004). 15273688 10.1038/ng1399

[b35] Bell A. C., West A. G. & Felsenfeld G. The protein CTCF is required for the enhancer blocking activity of vertebrate insulators. Cell 98, 387–396 (1999). 10458613 10.1016/s0092-8674(00)81967-4

[b36] Jungmichel S. . Proteome-wide identification of poly(ADP-Ribosyl)ation targets in different genotoxic stress responses. Molecular cell 52, 272–285 (2013). 24055347 10.1016/j.molcel.2013.08.026

[b37] Oberoi J. . Structural basis of poly (ADP-ribose) recognition by the multizinc binding domain of checkpoint with forkhead-associated and RING Domains (CHFR). Journal of Biological Chemistry 285, 39348–39358 (2010). 20880844 10.1074/jbc.M110.159855 PMC2998101

[b38] Nakahashi H. . A genome-wide map of CTCF multivalency redefines the CTCF code. Cell reports 3, 1678–1689 (2013). 23707059 10.1016/j.celrep.2013.04.024 PMC3770538

[b39] Lee B. K. & Iyer V. R. Genome-wide studies of CCCTC-binding factor (CTCF) and cohesin provide insight into chromatin structure and regulation. The Journal of biological chemistry 287, 30906–30913 (2012). 22952237 10.1074/jbc.R111.324962 PMC3438923

[b40] Sancar A., Lindsey-Boltz L. A., Ünsal-Kaçmaz K. & Linn S. Molecular mechanisms of mammalian DNA repair and the DNA damage checkpoints. Annual review of biochemistry 73, 39–85 (2004). 10.1146/annurev.biochem.73.011303.073723 15189136

[b41] Fahrer J., Kranaster R., Altmeyer M., Marx A. & Bürkle A. Quantitative analysis of the binding affinity of poly (ADP-ribose) to specific binding proteins as a function of chain length. Nucleic acids research 35, e143–e143 (2007). 17991682 10.1093/nar/gkm944 PMC2175335

