# Supplementary material for: CTCF participates in DNA damage response via poly(ADP-ribosyl)ation
Source: Sci Rep. 2017 Mar 6;7:43530. doi: 10.1038/srep43530 (PMC5337984; doi:10.1038/srep43530)
Supplement: Supplemental Data [file srep43530-s1.pdf]

Supplementary Information

CTCF participates in DNA damage response via poly(ADP-ribosyl)ation

Deqiang Han, Qian Chen, Jiazhong Shi, Feng Zhang and Xiaochun Yu

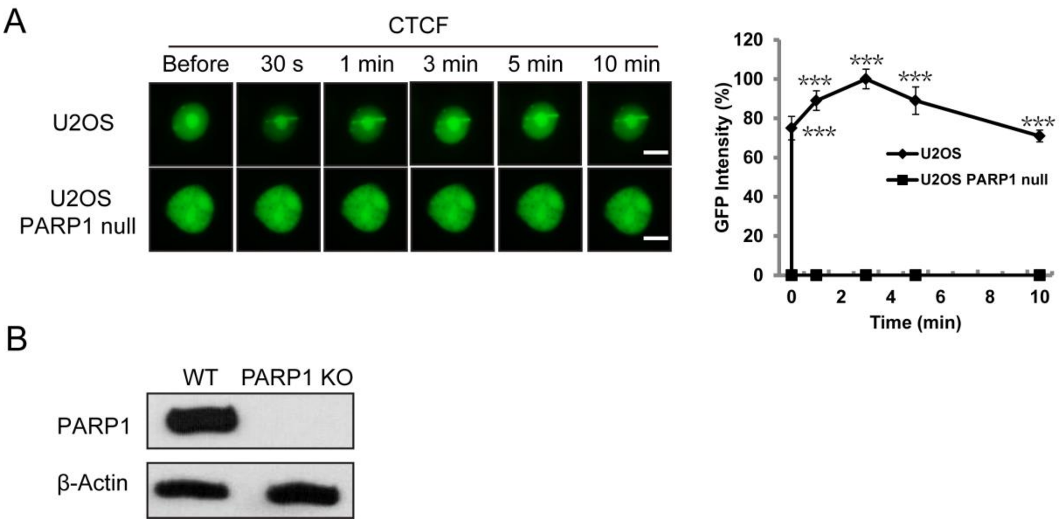

Figure S1 The recruitment of CTCF to DNA damage site was disrupted in the PARP1 deficient U2OS cells. (A) GFP-tagged CTCF was expressed in U2OS cells or PARP1 knockout U2OS cells, and the relocation was monitored in a time course following laser microirradiation. GFP signal intensities at the laser lines were converted into a numerical value using Image J software. Normalized fluorescent curves from 20 cells were averaged. Scale bar = 10 μm. The error bars represent the standard deviation. Significance of differences was evaluated by Student's t test. *** $p < 0.001$. (B) The expression levels of PARP1 in two cell lines were identified by western blotting.

Supplementary Information

CTCF participates in DNA damage response via poly(ADP-ribosyl)ation

Deqiang Han, Qian Chen, Jiazhong Shi, Feng Zhang and Xiaochun Yu

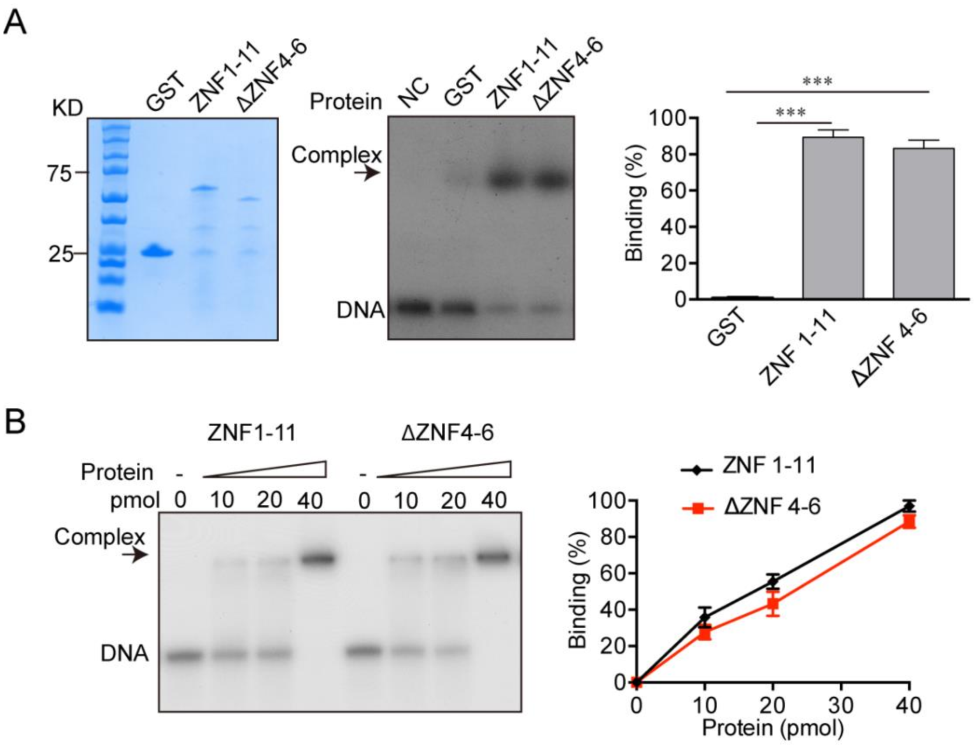

Figure S2 PAR-binding module of CTCF is dispensable for DNA binding. (A) Left: Gel mobility shift DNA binding analysis of ZNF 1-11(lane 3) and ΔZNF4-6 (lane 4), whereas GST (lane 2), and PBS (lane 1) were negative controls. Middle panel: SDS–PAGE of purified recombinant proteins. Purified proteins were loaded on a 10 % SDS–PAGE, and stained with coomassie blue. Lane1: molecular weight markers (Bio-Rad); lane 2: GST; lane 3: GST-ZNF1-11; lane 4: GST-ΔZNF4-6, Quantification of the blots in the left panel is shown on the right panel. The values are the average of three independent experiments. Error bars represent the SD. (B) ^{32}P labeled DNA was incubated together with an increasing concentration of ZNF1-11 (lane 1-4) and ΔZNF4-6 (lane 5-8). Quantification of the blots is shown on the right panel. The arrowheads indicate the protein-DNA complex. Error bars represent the SD. Significance of differences was evaluated by Student’s t test. *** $p < 0.001$.

Supplementary Information

CTCF participates in DNA damage response via poly(ADP-ribosyl)ation

Deqiang Han, Qian Chen, Jiazhong Shi, Feng Zhang and Xiaochun Yu

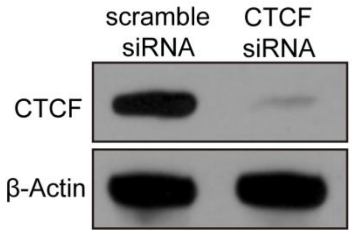

Figure S3 The expression level of CTCF protein was dramatically reduced with the siRNA knockdown. Western blotting was performed using anti-CTCF antibody. β-actin was used for protein loading control. The CTCF blot is cropped and the full-length blot is included in the Figure S5B.

Supplementary Information

CTCF participates in DNA damage response via poly(ADP-ribosyl)ation

Deqiang Han, Qian Chen, Jiazhong Shi, Feng Zhang and Xiaochun Yu

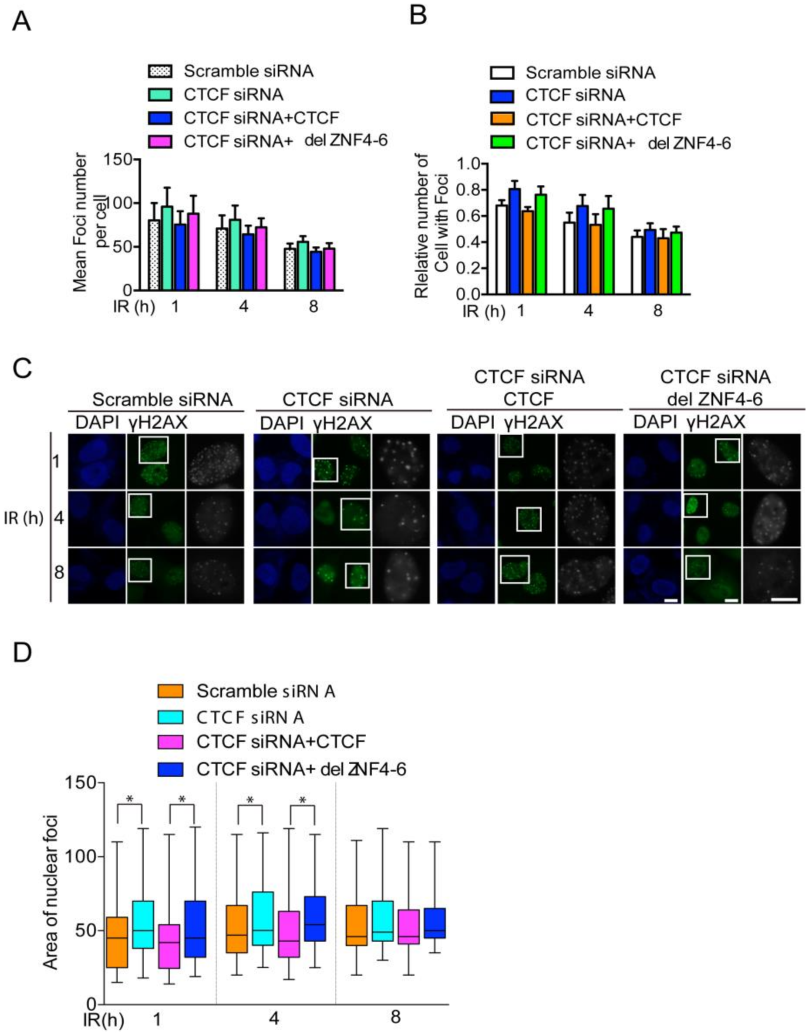

Figure S4 The possible role of CTCF to set up the boundary for DNA damage response (A) Number of γH2AX IRIF in each cell from CTCF-depleted cells with or without reconstituting plasmids. The cells were irradiated with 8 Gy IR, fixed and immunostained with γH2AX antibody at different time points. The number of γH2AX foci was counted using ImageJ software. 100 cells were analyzed. Box plots and statistical analysis were performed using GraphPad Prism 5. Error bars represent the SD. Significance of differences was evaluated by Student's t test. (B) Number of cell with IRIF was analyzed as (A). (C)

Images of γH2AX IRIF from CTCF-depleted cells at different time points following 8 Gy of IR. (D) The size of IRIF of γH2AX was measured by Image J software. Box plots and statistical analysis were performed using GraphPad Prism 5, Error bars represent the SD, n=3. Significance of differences was evaluated by Student's t test. * $p < 0.05$.

Supplementary Information

CTCF participates in DNA damage response via poly(ADP-ribosyl)ation

Deqiang Han, Qian Chen, Jiazhong Shi, Feng Zhang and Xiaochun Yu

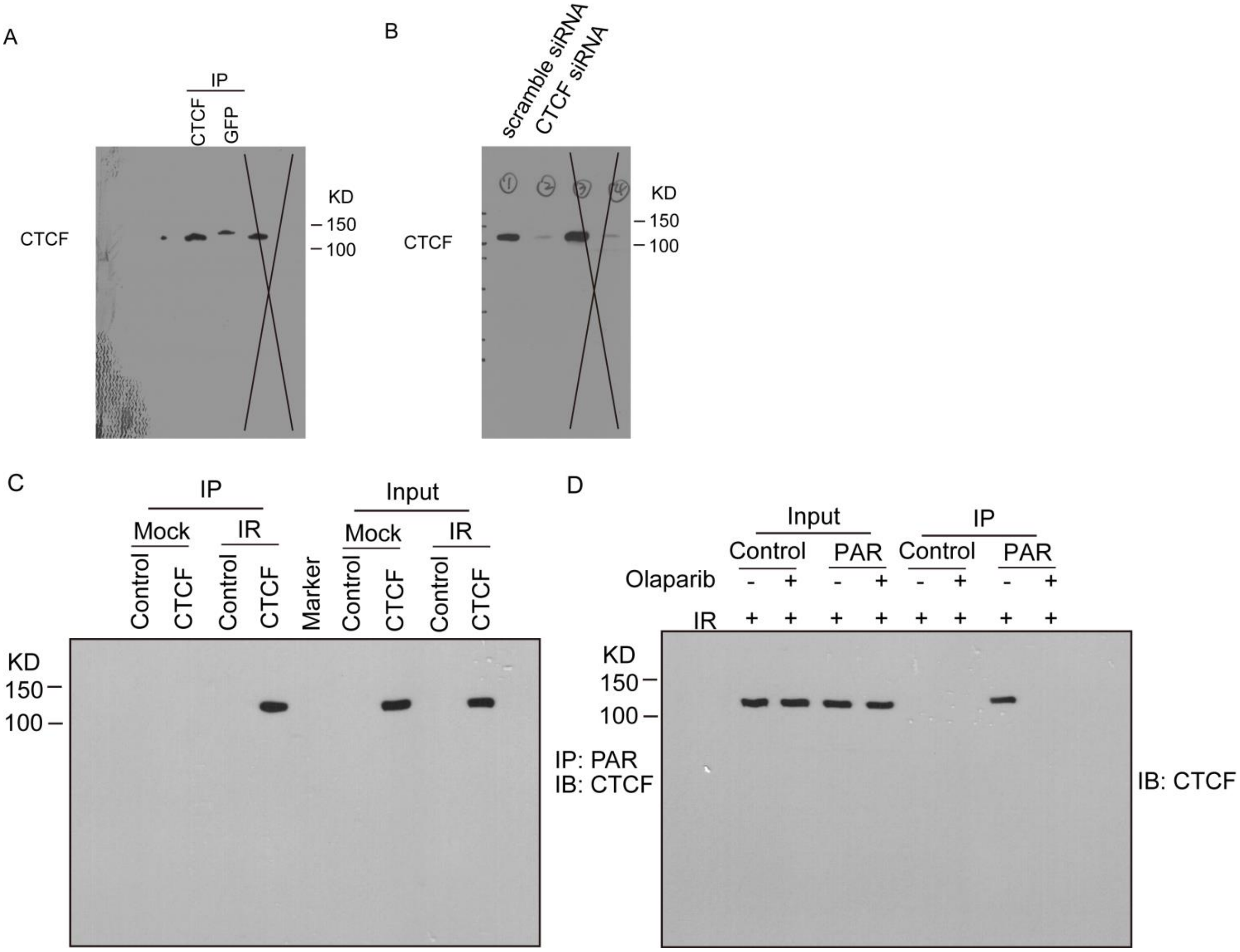

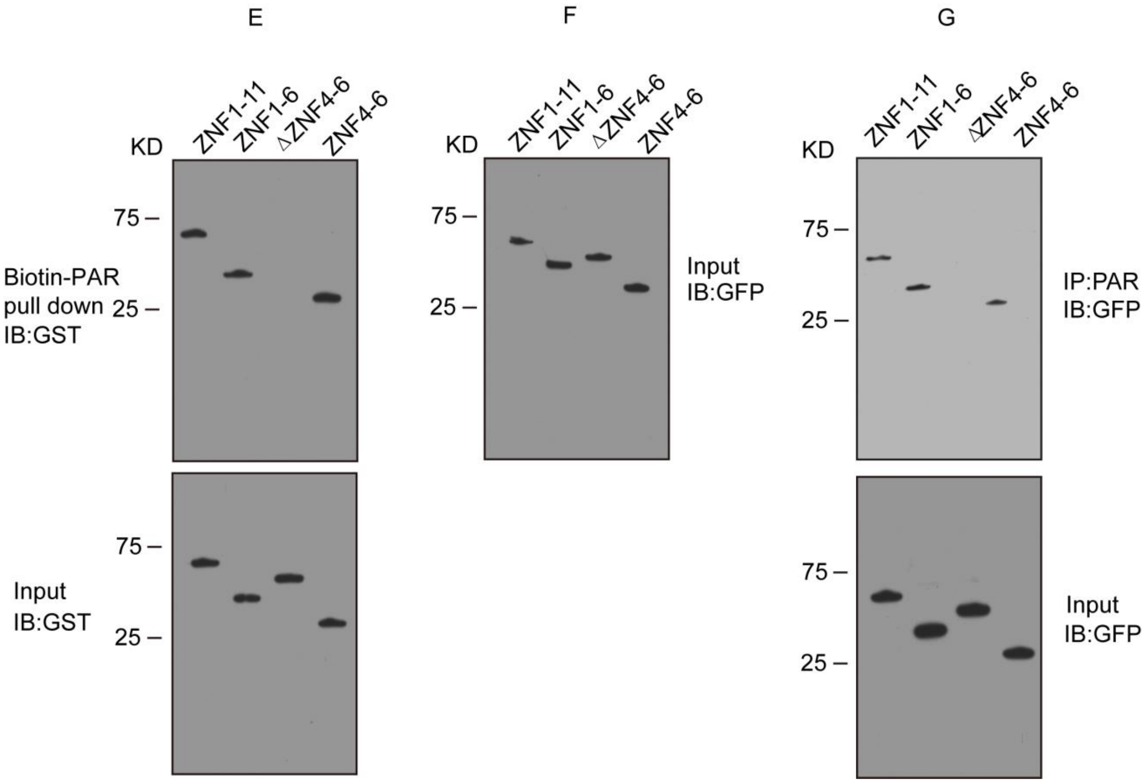

Figure S5 The full-length blots (A) Full-length blot for original Fig 1B (B) Full-length blot for original supplemental figure 3. (C) Full-length blot for original Fig 3D. (D) Full-length blot for original figure 3F. (E) Full-length blot for original Fig 5B. (F) Full-length blot for original figure 5C. (G) Full-length blot for original Fig 5D.
